# Impact of previous abdominal surgery on robotic-assisted rectal surgery in patients with locally advanced rectal adenocarcinoma: a propensity score matching study

**DOI:** 10.1186/s12957-020-02086-1

**Published:** 2020-11-25

**Authors:** Ching-Wen Huang, Wei-Chih Su, Tsung-Kun Chang, Cheng-Jen Ma, Tzu-Chieh Yin, Hsiang-Lin Tsai, Po-Jung Chen, Yen-Cheng Chen, Ching-Chun Li, Yi-Chien Hsieh, Jaw-Yuan Wang

**Affiliations:** 1 Division of Colorectal Surgery, Department of Surgery, Kaohsiung Medical University Hospital, Kaohsiung Medical University, 100 Tzyou 1st Road, Kaohsiung, 807 Taiwan; 2 Department of Surgery, Faculty of Medicine, College of Medicine, Kaohsiung Medical University, Kaohsiung, Taiwan; 3 Division of General and Digestive Surgery, Department of Surgery, Kaohsiung Medical University Hospital, Kaohsiung Medical University, Kaohsiung, Taiwan; 4 Department of Surgery, Kaohsiung Municipal Tatung Hospital, Kaohsiung Medical University, Kaohsiung, Taiwan; 5 Division of Colorectal Surgery, Department of Surgery, Kaohsiung Municipal Hsiaokang Hospital, Kaohsiung, Taiwan; 6 Graduate Institute of Clinical Medicine, College of Medicine, Kaohsiung Medical University, Kaohsiung, Taiwan; 7 Graduate Institute of Medicine, College of Medicine, Kaohsiung Medical University, Kaohsiung, Taiwan; 8 Center for Cancer Research, Kaohsiung Medical University, Kaohsiung, Taiwan; 9 Center for Liquid Biopsy and Cohort Research, Kaohsiung Medical University, Kaohsiung, Taiwan; 10 Master Program for Clinical Pharmacogenomics and Pharmacoproteomics, School of Pharmacy, Taipei Medical University, Taipei, Taiwan

**Keywords:** Pervious abdominal surgery, Robotic-assisted rectal surgery, Locally advanced rectal adenocarcinoma, Propensity score matching

## Abstract

**Background:**

The application of minimally invasive surgery in patients with colorectal cancer (CRC) and a history of previous abdominal surgery (PAS) remains controversial. This retrospective study with propensity score matching (PSM) investigated the impact of PAS on robotic-assisted rectal surgery outcomes in patients with locally advanced rectal adenocarcinoma undergoing preoperative concurrent chemoradiotherapy (CCRT).

**Methods:**

In total, 203 patients with locally advanced rectal adenocarcinoma who underwent preoperative CCRT and robotic-assisted rectal surgery between May 2013 and December 2019 were enrolled. Patients were categorized into PAS and non-PAS groups based on the PAS history. The PSM caliper matching method with 1-to-3 matches was used to match PAS patients with non-PAS.

**Results:**

Of the 203 enrolled patients, 35 were PAS patients and 168 were non-PAS patients. After PSM, 32 PAS patients and 96 non-PAS patients were included for analysis. No significant between-group differences were noted in the perioperative outcomes, including median console time (165 min (PAS) vs. 175 min (non-PAS), *P* = 0.4542) and median operation time (275 min (PAS) vs. 290 min (non-PAS), *P* = 0.5943) after PSM. Postoperative recovery and overall complication rates were also similar (all *P* > 0.05). Moreover, the between-group differences in pathological or short-term oncological outcomes were also nonsignificant (all *P* > 0.05). No 30-day postoperative deaths were observed in either group.

**Conclusion:**

The current results indicate that robotic-assisted surgery is safe and feasible for PAS patients with locally advanced rectal adenocarcinoma undergoing preoperative CCRT. However, future prospective randomized clinical trials are required to verify these findings.

## Background

According to the last issue of GLOBOCAN (2018), colorectal cancer (CRC) is the third most common cancer and the third leading cause of cancer-related deaths in the world [1]. In 2017, approximately 1.8 million new CRC diagnoses and 896,000 CRC-related deaths were reported globally [2]. In Taiwan, CRC is the most common cancer type and has been the third leading cause of cancer-related deaths since 1996. In 2000 and 2017, its incidence was 32.38 and 66.32 per 100,000, respectively (with 7213 and 16,408 new diagnoses, respectively). Moreover, in 2017, there were 10,209 new diagnoses of colon cancers and 6199 new diagnoses of rectal cancers in Taiwan. In 2019, 6436 people in Taiwan died of CRC, with the mortality rate being 27.3 and 20.6 per 100,000 individuals in 2019 and 2009, respectively [3].

Minimally invasive surgery with laparoscopic access provides several benefits compared with open surgery, such as less postoperative pain, early mobilization, earlier postoperative recovery, and shorter hospital stay [4–7]. Moreover, oncological outcomes were similar between laparoscopic and open surgical procedures [4–8]. Robotic-assisted surgery offers numerous advantages, such as high-definition three-dimensional vision with up to 10× magnification, articulatory instruments, a surgeon-controlled camera platform, and stable traction by the robot arms. Compared with conventional laparoscopic and open surgical procedures for rectal cancers, the clinical and short-term oncological outcomes of robotic-assisted surgery seem to be more favorable [9–12].

In general, majorities of intraabdominal adhesions result from abdominal surgeries [13]. In laparoscopic surgical procedures, intraabdominal adhesion may increase the difficulties of surgery and the risks of perioperative complications, including injury to intraperitoneal viscera and vascular structures [14]. In laparoscopic surgical procedures, disadvantages associated with intraabdominal adhesion are change of planned trocar sites, distortion of normal anatomy, limited vision, and low haptic sensation [15]. Laparoscopic surgical procedures in patients with intraabdominal adhesion lead to a higher conversion rate to open surgery and longer operation time. However, the advancements in laparoscopy instrumentation and increases in the relevant experience have made the application of laparoscopic colorectal surgery feasible and safe for patients with previous abdominal surgery (PAS) [14–19]. Nevertheless, Yamamoto et al. [20] reported significantly longer postoperative recovery time and higher rate of inadvertent enterotomy as well as more frequent ileus in PAS patients. Therefore, the application of minimally invasive surgery in intraabdominal adhesion-complicated PAS patients with CRC is a matter of contention [14, 16, 20]. Few studies have also evaluated the impact of PAS on robotic-assisted colorectal surgery in patients with CRC [18, 21]. Here, we conducted a retrospective study with propensity score matching (PSM) to investigate the effect of PAS on robotic-assisted rectal surgery in patients with locally advanced rectal cancer undergoing preoperative concurrent chemoradiotherapy (CCRT).

## Materials and methods

### Patients

**Fig. 1:**
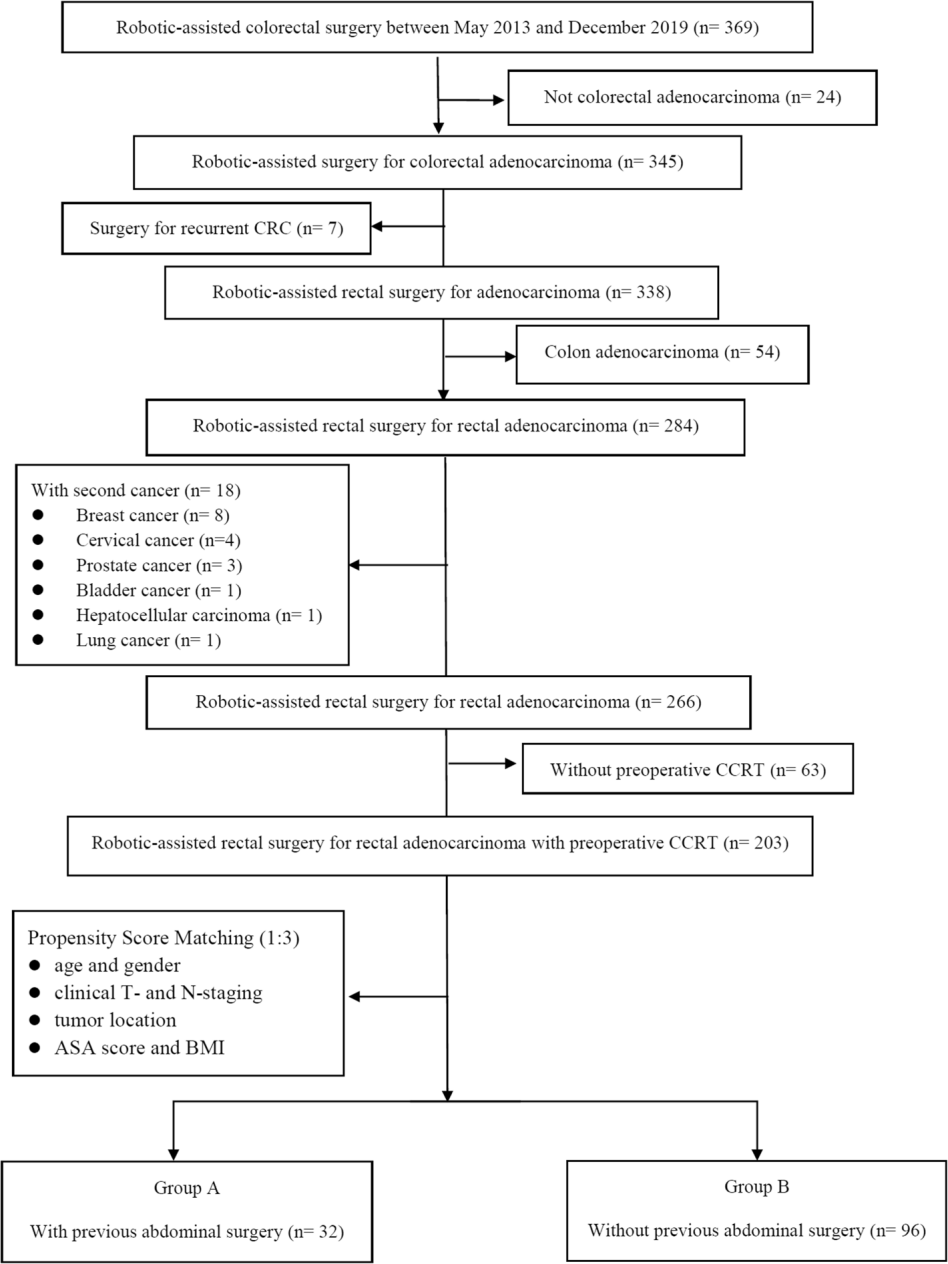
CONSORT diagram showing the inclusion and exclusion criteria

We retrospectively analyzed data from Kaohsiung Medical University Hospital in Taiwan. The inclusion criteria were histologically proven rectal adenocarcinoma with the tumor located within 15 cm from the anal verge, preoperative CCRT with long-course radiotherapy (LCRT), and robotic-assisted surgery. The exclusion criteria were surgeries for recurrent cancer and presence of a second primary cancer. In total, 203 consecutive patients meeting the inclusion criteria underwent preoperative CCRT followed by robotic-assisted mesorectal excision (TME) with the single-docking technique [22] using the da Vinci® Si surgical system (Intuitive Surgical, Inc., Sunnyvale, CA, USA) between September 2013 and February 2019 (Fig. 1). This study was approved by the Institutional Review Board of Kaohsiung Medical University Hospital (KMUHIRB-E(I)-20200036).

Accordingly, preoperative staging studies included colonoscopy and abdominal and pelvic computed tomography (CT) or high-definition magnetic resonance imaging (MRI), and chest radiological assessment in all patients. Based on the distance from the anal verge, rectal cancer was categorized into upper (11–15 cm), middle (6–10 cm), and lower (≤ 5 cm). Patients with locally advanced rectal cancer (LARC, i.e., T3, T4, or N+ rectal cancer) underwent preoperative CCRT, which was a FOLFOX (i.e., 5-fluorouracil, leucovorin, and oxaliplatin) regimen every 2 weeks and LCRT (total of 5000 cGy in 25 fractions), as described previously [23]. Each cycle of FOLFOX included oxaliplatin (85 mg/m^2^) on day 1, folic acid (400 mg/m^2^) on day 1, and a 46-hour infusion of 5-FU (2800 mg/m^2^) [23]. Patients with cT2 rectal cancer located within 5 cm from the anal verge also underwent the same preoperative CCRT. After radiotherapy, all patients continuously underwent chemotherapy of the same FOLFOX regimen up to 2–3 weeks before robotic-assisted rectal surgery. Accordingly, abdominal and pelvic CT or high-definition MRI was performed for restaging of rectal cancer. If the rectal cancer was resectable, robotic-assisted TME was performed using the single-docking technique [22]. During surgery, laparoscopic adhesiolysis was performed first if peritoneal adhesion was noted before the robotic-assisted rectal surgery.

The following clinicopathological features and perioperative parameters were evaluated: age, sex, TNM (tumor, node, and metastasis) classification, histological type, perineural invasion, vascular invasion, postoperative serum carcinoembryonic antigen (CEA) levels, tumor location (distance from the anal verge), body mass index (BMI), and American Society of Anesthesiologists (ASA) score. The TNM classification was determined according to the criteria of the 7th edition of the American Joint Commission on Cancer (AJCC) [24]. The tumor regression grade (TRG) was determined according to the AJCC and College of American Pathologists regression grade [24, 25]. Perioperative outcomes, including surgical procedures, docking time, operation time, console time, estimated blood loss, duration of the first flatus passage after surgery, duration for resuming a soft diet after surgery, duration of postoperative hospital stay, and postoperative first day visual analog scale (VAS) pain score, were evaluated.

After robotic-assisted surgery, adjuvant chemotherapy was administrated, as reported previously [23]. In brief, an additional 5–6 cycles of the FOLFOX regimen were administrated every 2 weeks (12 perioperative cycles in total) for patients with the following risk factors: (1) ypN1–2, (2) positive circumferential resection margin (CRM) or distal resection margin (DRM), and (3) ypT3–4. For patients with ypT1–2 N0 rectal cancers, fluoropyrimidine-based chemotherapy was administrated for up to 6 months of perioperative chemotherapy. Patients were followed up regularly, with their clinical outcomes and survival statuses being recorded, as described previously [23].

### Statistical analysis

To reduce the potential selection bias, we used PSM to match the compatible groups. The PSM caliper matching method with 1-to-3 matches was used to match PAS patients with non-PAS patients without. The covariates included patient demographic characteristics (age and sex), clinical cancer stage (including T- and N-staging), tumor location (distance from the anal verge), ASA score, and BMI. All data were fully anonymized before they were accessed. All data were statistically analyzed using the Statistical Analysis System (SAS) software, version 9.4 (SAS Institute Inc., SAS Campus Drive, Cary, North Carolina 27513, USA). All patients were followed up regularly until their death or last follow-up, whichever occurred first. The docking time was defined as the time required to place the robot and make sure of the robot arms to the corresponding port sites. The console time was defined as the total duration of robotic-assisted surgical procedures with the robotic system (da Vinci® Si surgical system). The operation time was defined as the total duration between the initial skin incision and the completion of wound closure. The correlation between clinicopathological features and treatment groups was examined using the chi-square test for categorical variables and Student *t* test for continuous variables. A *P* value of < 0.05 indicated statistical significance. Disease-free survival (DFS) was defined as the duration between the date of primary treatment and the date of diagnosis of recurrent or metastatic disease or the date of last follow-up. Overall survival (OS) was defined as the duration between the date of primary treatment and the date of death from any cause or last follow-up. DFS and OS were evaluated using the Kaplan–Meier method, and the log-rank test was performed to compare time-to-event distributions. DFS and OS were evaluated using the Kaplan–Meier method, and the log-rank test was used to compare time-to-event distributions. A *P* < 0.05 was considered statistically significant.

## Results

### Patient characteristics and perioperative outcomes

Between May 2013 and December 2019, 369 patients underwent robotic-assisted surgery. Of these patients, 203 who met the including criteria and exclusion criteria were enrolled in this study. Of them, 35 had a history of PAS, whereas 168 did not. After PSM, 32 PAS and 96 non-PAS patients were included for analysis (Fig. 1). The baseline characteristics and perioperative outcomes of the patients before (*n* = 203) and after (*n* = 128) PSM are summarized in Table 1.

**Table 1:** Baseline characteristics

Characteristic	Overall					After match (1:3)				
	With PAS (N=35)		Without PAS (N=168)		***p*** value	With PAS (N=32)		Without PAS (N=96)		***p*** value
	N	%	N	%		N	%	N	%	
Age (Median, range) (years)	61.0 (38.0–83.0)		61.0 (28.0–93.0)		0.4270	61.0 (38.0–83.0)		62.5 (31.0–93.0)		0.5986
Gender					0.0006^*^					0.7964
Female	22	62.9%	54	32.1%		19	59.4%	55	57.3%	
Male	13	37.1%	114	67.9%		13	40.6%	41	42.7%	
Tumor distance from anal verge (cm)					0.8960					0.7280
≦5 (Lower)	19	54.3%	93	55.4%		18	56.3%	54	56.3%	
6–10 (Middle)	12	34.3%	52	31.0%		10	31.3%	25	26.0%	
11–15 (Upper)	4	11.4%	23	13.7%		4	12.5%	17	17.7%	
Distance from anal verge (cm)										
Median (range)	5.0 (0.5–15.0)		5.0 (0.5–20.0)		0.7624	5.0 (1.5–15.0)		5.0 (0.5–20.0)		0.8430
Mean ± SD	6.5±4.0		6.4±4.1		0.8598	6.5±4.0		6.6±4.7		0.9263
Post-op serum CEA level					0.7429					0.7645
<5 ng/ml	29	90.6%	151	91.5%		27	93.1%	85	91.4%	
≥5 ng/ml	3	9.4%	14	8.5%		2	6.9%	8	8.6%	
ASA score					0.9054					0.4656
2	21	60.0%	102	61.1%		21	65.6%	56	58.3%	
3	14	40.0%	65	38.9%		11	34.4%	40	41.7%	
BMI kg/m2										
Median (range)	23.6 (18.7–27.5)		23.4 (17.4–37.8)		0.4217	23.5 (18.7–27.5)		22.7 (17.6–30.2)		0.8994
Mean ± SD	23.4±2.9		24.1±3.5		0.2585	23.1±2.9		23.3±3.0		0.8039
Perioperative outcomes										
Procedure					0.7273					0.8289
LAR	22	62.9%	103	61.3%		20	62.5%	58	60.4%	
ISR	12	34.3%	63	37.5%		12	37.5%	37	38.5%	
APR	1	2.9%	2	1.2%		0	0.0%	1	1.0%	
Protective Diverting Colostomy					0.2587					0.4444
Yes	14	41.2%	86	51.8%		13	40.6%	46	48.4%	
No	20	58.8%	80	48.2%		19	59.4%	49	51.6%	
Protective Diverting Colostomy in LAR					0.2409					0.2505
Yes	2	9.1%	23	22.3%		1	5.0%	9	14.8%	
No	20	90.9%	80	77.7%		19	95.0%	49	85.2%	
Docking Time (minutes)										
Median (range)	3 (3–8)		4 (2–11)		0.0733	3 (3–8)		4 (2–11)		0.0956
Mean ± SD	3.9±1.3		4.4±1.6		0.1098	3.9±1.3		4.3±1.5		0.2192
Console Time (minutes)										
Median (range)	167.5 (120–240)		187.5 (95–374)		0.0394*	165 (120–240)		175 (95–305)		0.4542
Mean ± SD	172.9±36.4		194.0±53.3		0.0062*	172.7±37.2		180.3±45.4		0.3974
Operation Time (minutes)										
Median (range)	275 (210–430)		300 (180–855)		0.0748	275 (210–430)		290 (200–620)		0.5943
Mean ± SD	290.9±50.9		326.2±100.4		0.0035*	290.7±51.3		305.4±84.0		0.2517
Estimated blood loss (mL)										
Median (range)	65 (15–350)		70 (15–1050)		0.3776	50 (15–350)		50 (20–700)		0.9424
Mean ± SD	88.3 ±71.4		119.6 ±141.8		0.0667	83.1±71.2		85.1±102.8		0.9054
Time of first flatus passage (day)										
Median (range)	2 (1–3)		2 (0–10)		0.5821	2 (1–3)		1 (1–10)		0.5190
Mean ± SD	1.7±0.7		1.7±1.0		0.8980	1.6±0.7		1.6±1.1		0.8872
Time of resuming soft diet (day)										
Median (range)	4 (2–5)		3 (2–15)		0.7415	4 (2–5)		3 (2–15)		0.6076
Mean ± SD	3.8±0.8		3.8±1.7		0.2177	3.5±0.7		3.8±1.9		0.2710
Postoperative hospital stay (day)										
Median (range)	7 (5–46)		6 (4–32)		0.0676	7 (5–46)		6 (4–18)		0.1178
Mean ± SD	8.0±6.8		7.1±3.0		0.4109	8.1±7.1		6.8±2.3		0.3274
Postoperative first day pain score										
Median (range)	3 (0–8)		3 (0–8)		0.8384	3 (0–8)		3 (0–7)		0.8329
Mean ± SD	3.4±1.9		3.2±1.5		0.6549	3.2±1.7		3.0±1.4		0.5675

^*^P value < 0.05

*PAS* previous abdominal surgery, *SD* standard deviation, *CEA* carcinoembryonic antigen, *ASA* American Society of Anesthesiologist, *BMI* body mass index, *LAR* low anterior resection, *ISR* intersphincteric resection, *APR* abdominoperineal resection

Before PSM, the between-group differences in sex (*P* = 0.006) and console time (*P* = 0.0394) were significant. Moreover, before PSM, the median console time was shorter in PAS patients than in non-PAS patients (167.5 vs. 187.5 min, *P* = 0.0394), but no such between-group difference in median operation times (275 min (PAS) vs. 300 min (non-PAS), *P* = 0.0748). After PSM, both median console times were similar between the two groups (165 min (PAS) vs. 175 min (non-PAS), *P* = 0.4542), and the median operation times (275 min (PAS) vs. 290 min (non-PAS), *P* = 0.5943) were similar between the groups. With the PSM, the comparison between the groups was adjusted for confounding variables, and this analysis showed that there is no difference between the groups.

### Pathological and oncological outcomes

Table 2 summarizes the pathological characteristics and oncological outcomes of the patients before (*n* = 203) and after (*n* = 128) PSM. Preoperative clinical staging demonstrated that most patients with LARC had T3 lesions: 27 (77.1%) PAS and 129 (76.8%) non-PAS patients. Even after PSM, most patients with LARC had T3 lesions: 25 (77.1%) PAS and 74 (77.1%) non-PAS, respectively. No significant differences were observed in terms of clinical T, N, and AJCC stages in PAS and non-PSA patients (all *P* > 0.05). Moreover, no significant differences were observed in terms of postoperative pathological and oncological outcomes (all *P* > 0.05). Furthermore, no significant differences were observed in terms of preoperative clinical staging, postoperative pathological outcomes, and oncological outcomes (all *P* > 0.05) after PSM.

**Table 2:** Pathologic characteristics and oncological outcomes

Characteristic	Overall						After match (1:3)			
	With PAS (*N* = 35)		Without PAS (*N* = 168)		*P* value	With PAS (*N* = 32)		Without PAS (*N* = 96)		*P* value
	*N*	%	*N*	%		*N*	%	*N*	%	
Preoperative clinical staging										
Tumor depth					0.9823					0.9270
T2	2	5.7%	11	6.5%		1	3.1%	5	5.2%	
T3	27	77.1%	129	76.8%		25	78.1%	74	77.1%	
T4	6	17.1%	28	16.7%		6	18.8%	17	17.7%	
Lymph node metastasis					0.2970					0.9345
N0	11	31.4%	33	19.6%		8	25.0%	26	27.1%	
N1	15	42.9%	88	52.4%		15	46.9%	46	47.9%	
N2	9	25.7%	47	28.0%		9	28.1%	24	25.0%	
AJCC stage (clinical)					0.3444					0.9846
I	2	5.7%	4	2.4%		1	3.1%	4	4.2%	
II	7	20.0%	26	15.5%		7	21.9%	21	21.9%	
III	20	57.1%	120	71.4%		20	62.5%	59	61.5%	
IV	6	17.1%	18	10.7%		4	12.5%	12	12.5%	
Postoperative pathological outcomes										
Tumor size					0.3634					0.2124
< 5 cm	35	100.0%	159	94.6%		32	100.0%	92	95.8%	
≥ 5 cm	0	0.0%	9	5.4%		0	0.0%	4	4.2%	
Tumor size (cm)										
Median (range)	1.7 (0–4.5)		1.7 (0–8.0)		0.8658	1.8 (0–4.5)		1.5 (0–8.0)		0.4810
Mean ± SD	1.8 ± 1.2		1.9 ± 1.7		0.6117	1.9 ± 1.2		1.7 ± 1.7		0.6547
Tumor depth					0.6850					0.3077
T0	7	20.0%	47	28.0%		6	18.8%	28	29.2%	
Tis	0	0.0%	2	1.2%		0	0.0%	2	2.1%	
T1	3	8.6%	11	6.5%		3	9.4%	8	8.3%	
T2	9	25.7%	43	25.6%		7	21.9%	26	27.1%	
T3	16	45.7%	60	35.7%		16	50.0%	29	30.2%	
T4	0	0.0%	5	3.0%		0	0.0%	3	3.1%	
Lymph node metastasis					0.8948					0.8281
N0	27	77.1%	126	75.0%		24	75.0%	72	75.0%	
N1	7	20.0%	32	19.0%		7	21.9%	18	18.8%	
N2	1	2.9%	9	5.4%		1	3.1%	5	5.2%	
N3	0	0.0%	1	0.6%		0	0.0%	1	1.0%	
AJCC stage (pathologic)					0.5986					0.7447
0	6	17.1%	45	26.8%		6	18.8%	27	28.1%	
I	10	28.6%	36	21.4%		9	28.1%	23	24.0%	
II	8	22.9%	35	20.8%		8	25.0%	16	16.7%	
III	5	14.3%	32	19.0%		5	15.6%	17	17.7%	
IV	6	17.1%	20	11.9%		4	12.5%	13	13.5%	
Down stage of T stage					0.4106					0.4188
Down stage	23	65.7%	116	69.0%		21	65.6%	72	75.0%	
Unchanged	12	34.3%	46	27.4%		11	34.4%	23	24.0%	
Up stage	0	0.0%	6	3.6%		0	0.0%	1	1.0%	
Down stage of N stage					0.6294					0.7574
Down stage	21	60.0%	112	66.7%		21	65.6%	57	59.4%	
Unchanged	13	37.1%	49	29.2%		10	31.3%	34	35.4%	
Up stage	1	2.9%	7	4.2%		1	3.1%	5	5.2%	
Down stage of AJCCstage					0.4548					0.8358
Down stage	19	54.3%	110	65.5%		19	59.4%	62	64.6%	
Unchanged	15	42.9%	54	32.1%		12	37.5%	31	32.3%	
Up stage	1	2.9%	4	2.4%		1	3.1%	3	3.1%	
Tumor regression grade					0.4980					0.3177
0	7	20.6%	46	28.6%		6	19.4%	27	30.3%	
1	14	41.2%	68	42.2%		13	41.9%	39	43.8%	
2	11	32.4%	34	21.1%		10	32.3%	16	17.4%	
3	2	5.9%	13	8.1%		2	6.5%	8	8.4%	
Tumor regression					0.4992					0.1747
Good (0 + 1)	21	61.8%	114	70.8%		19	61.3%	66	74.2%	
Poor (2 + 3)	13	38.2%	47	29.2%		12	38.7%	23	25.8%	
Harvested lymph node										
Median (range)	10 (2–33)		9 (0–36)		0.7059	10 (2–23)		9 (1–30)		0.2360
Mean ± SD	10.7 ± 5.8		10.3 ± 5.7		0.6939	11.2 ± 5.8		9.7 ± 5.5		0.2020
Positive lymph node										
Median (range)	0 (0–6)		0 (0–24)		0.6550	0 (0–6)		0 (0–24)		0.7394
Mean ± SD	0.4 ± 1.1		0.6 ± 2.3		0.3068	0.4 ± 1.2		0.8 ± 3.0		0.3348
Harvested apical node										
Median (range)	2 (0–10)		2 (0–10)		0.6824	2 (0–10)		2 (0–8)		0.9269
Mean ± SD	2.3 ± 2.1		2.4 ± 2.1		0.7417	2.3 ± 2.1		2.2 ± 1.8		0.7394
Positive apical node										
Median (range)	0 (0–3)		0 (0–2)		0.2219	0 (0–3)		0 (0–2)		0.3176
Mean ± SD	0.2 ± 0.6		0.1 ± 0.3		0.2939	0.2 ± 0.6		0.1 ± 0.4		0.3540
Vascular invasion					1.0000					0.9158
No	31	91.2%	143	91.7%		28	90.3%	81	91.0%	
Yes	3	8.8%	13	8.3%		3	9.7%	8	8.99%	
Perineural invasion					0.6028					0.8250
No	29	85.3%	128	81.5%		26	83.9%	74	82.22%	
Yes	5	14.7%	29	18.5%		5	16.1%	16	17.78%	
Distance of PRM (cm)										
Median (range)	5.0 (1.0–10.0)		5.5 (0.8–58.0)		0.3476	5.0 (1.0–10.0)		5.8 (0.8–58.0)		0.0924
Mean ± SD	5.2 ± 1.8		6.1 ± 4.9		0.0813	5.2 ± 1.8		7.0 ± 7.5		0.0412
Distance of DRM (cm)										
Median (range)	2.1 (0.1–8.1)		2 (0.1–7.5)		0.7923	2.4 (0.1–8.1)		2.0 (0.1–7.0)		0.5231
Mean ± SD	2.4 ± 1.9		2.3 ± 0.5		0.6451	2.4 ± 1.9		2.2 ± 1.5		0.4101
Distance of CRM (mm)										
Median (range)	11.5 (2.0–120.0)		12.0 (1.0–120.0)		0.9867	10.0 (2.0–120.0) 22.9		15.0 (1.0–98.0)		0.5309
Mean ± SD	23.0 ± 29.5		18.9 ± 20.9		0.4994	± 30.7		20.2 ± 20.5		0.6971
DRM					0.1383					0.3322
Free	33	94.3%	166	98.8%		30	93.8%	93	97.4%	
Positive	2	5.7%	2	1.2%		2	6.3%	3	2.6%	
CRM					1.0000					0.8828
Free	34	97.1%	161	97.0%		31	96.9%	93	96.9%	
Positive	1	2.9%	5	3.0%		1	3.1%	3	3.1%	
Resection degree of primary tumor					0.6549					0.8224
R0	33	94.3%	161	95.8%		30	93.8%	91	94.8%	
R1	2	5.7%	7	4.2%		2	6.3%	5	5.2%	
Oncological outcomes (clinical stages I–III)										
Follow-up periods (months)										
Median (range)	23.1 (8.4–74.6)		28.6 (6.2–87.1)		0.3183	22.5 (8.4–74.6)		30.5 (6.2–87.1)		0.4770
Mean ± SD	29.0 ± 16.5		32.9 ± 19.3		0.2579	29.4 ± 17.1		33.1 ± 19.7		0.3499
Post-op relapse					0.9904					0.8943
No	24	82.8%	124	82.7%		23	82.1%	70	83.3%	
Yes	5	17.2%	26	17.3%		5	17.9%	14	16.7%	
Post-op locoregional recurrent					0.5913					0.0828
No	28	96.6%	146	97.3%		27	96.4%	84	100.0%	
Yes	1	3.4%	4	2.7%		1	3.6%	0	0.0%	
Post-op distant metastasis					1.0000					0.7575
No	25	86.2%	128	85.3%		24	85.7%	70	83.3%	
Yes	4	13.8%	22	14.7%		4	14.3%	14	16.7%	
Death					1.0000					0.9377
No	31	88.6%	150	89.3%		28	87.5%	85	88.5%	
Yes	4	11.4%	18	10.7%		4	12.5%	11	11.5%	
DFS (months)										
Median (range)	21.3 (8.4–74.6)		24.4 (2.5–87.1)		0.7826	20.9 (8.4–74.6)		26.7 (2.5–87.1)		0.8103
Mean ± SD	27.6 ± 16.6		30.1 ± 19.7		0.5297	27.5 ± 16.9		29.8 ± 19.7		0.5773
OS (months)										
Median (range)	23.1 (8.4–74.6)		28.4 (6.4–87.1)		0.4110	22.5 (8.4–74.6)		30.7 (7.0–87.1)		0.4692
Mean ± SD	29.1 ± 16.8		32.8 ± 19.4		0.3292	29.4 ± 17.1		33.0 ± 19.6		0.3395

PAS previous abdominal surgery, *AJCC* American Joint Commission on Cancer, *SD* standard deviation, *PRM* proximal resection margin, *DRM* distal resection margin, *CRM* circumferential resection margin, *DFS* disease-free survival, *OS* overall survival

**Fig. 2:**
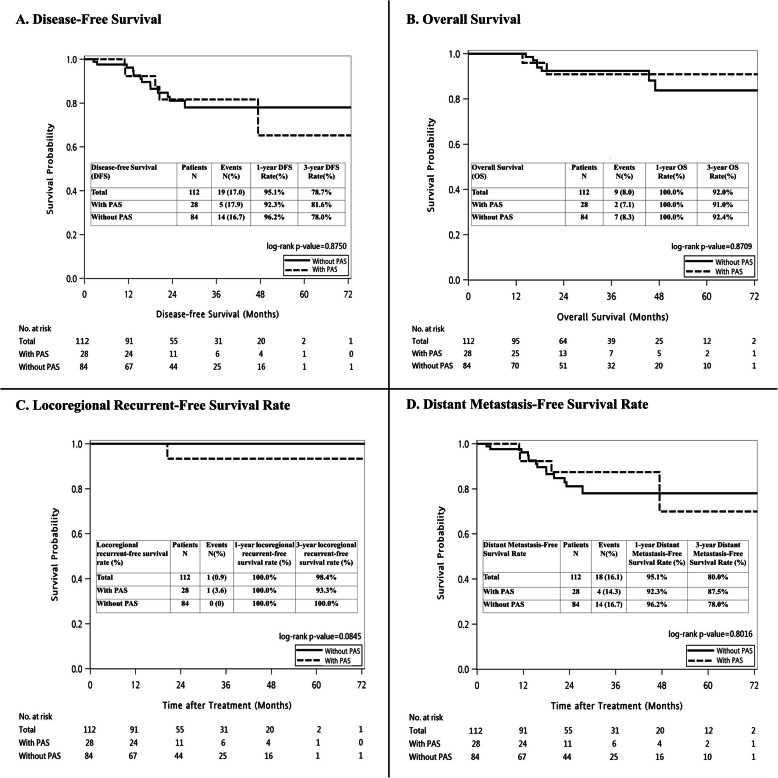
Kaplan–Meier survival curves for patients with stage I–III rectal cancer stratified by PAS history. **a** Disease-free survival. **b** Overall survival. **c** Locoregional recurrence-free survival. **d** Distant metastasis-free survival

After PSM, The median follow-up duration after primary treatment was 22.5 months (range: 8.4–74.6) and 30.5 months (range: 6.2–87.1 months) in PAS and non-PAS patients with stage I–III LARC, respectively. The median follow-up duration was not statistically different between the groups (*P* = 0.4770). Postoperative relapse was experienced by 5 (17.9%) PAS and 14 (16.7%) non-PAS patients, including local recurrence in 1 (3.6%) PAS patient, and distant metastases in 4 (14.3%) PAS and 14 (16.7%) non-PAS patients. Moreover, after PSM, PAS, and non-PAS patients demonstrated postoperative relapse, rates were comparable in patients with and without PAS after PSM, including overall, local recurrence, and distant metastasis rates (all *P* > 0.05). Furthermore, 4 (12.5%) PAS and 11 (11.5%) non-PAS patients died during follow-up (*P* = 0.9377). In PAS and non-PAS patients with stage I–III LARC, the 3-year DFS rates were 81.6% and 78.0%, respectively (Fig. 2a, *P* = 0.8750). Three-year OS rates were 91.0% and 92.4%, respectively (Fig. 2b, *P* = 0.8709); 3-year locoregional recurrence-free survival rates were 93.3% and 100.0%, respectively (Fig. 2c, *P* = 0.0845); 3-year distant metastasis-free survival rates were 87.5% and 78.0%, respectively (Fig. 2d, *P* = 0.8016).

### Postoperative complications

Postoperative complications in our PAS and non-PAS patients are summarized in Table 3. Postoperative complications were observed in 7 (21.9%) PAS and 19 (19.8%) non-PAS patients after PSM (*P* = 0.6010); however, the difference was nonsignificant (*P* = 0.6010). Moreover, anastomosis leakage was observed in 0 (0.0%) PAS and 4 (4.2%) non-PAS patients after PSM. Ileus was observed in 0 (0.0%) PAS and 4 (4.2%) non-PAS patients after PSM. Anastomosis stenosis was observed in 3 (9.4%) PAS and 2 (2.1%) non-PAS patients after PSM. Based on the Clavien-Dindo classification system, all postoperative complications were of grade I and the patients demonstrated an uneventful recovery course after conservative treatment. Moreover, no 30-day hospital mortality occurred.

**Table 3:** Postoperative complications

Complications	Overall					After match (1:3)				
	With PAS (*N* = 35)		Without PAS (*N* = 168)		*P* value	With PAS (*N* = 32)		Without PAS (*N* = 96)		*P* value
	*N*	%	*N*	%		*N*	%	*N*	%	
Total complications					0.9200					0.6010
No	28	80.0%	138	82.1%		25	78.1%	77	80.2%	
Yes	7	20.0%	30	17.9%		7	21.9%	19	19.8%	
Type of complications										
Anal bleeding	1	2.9%	1	0.6%		1	3.1%	0	0.0%	
Anastomosis stenosis	3	8.5%	5	3.0%		3	9.4%	2	2.1%	
Anastomosis leakage	0	0.0%	8	4.7%		0	0.0%	4	4.2%	
CVC infection	1	2.9%	0	0.0%		1	3.1%	0	0.0%	
Neck cellulitis	1	2.9%	0	0.0%		1	3.1%	0	0.0%	
Pulmonary complication	1	2.9%	2	1.2%		1	3.1%	3	3.1%	
Ileus	0	0.0%	6	3.6%		0	0.0%	4	4.2%	
Intraabdominal abscess	0	0.0%	3	1.8%		0	0.0%	1	1.0%	
Intraabdominal hematoma	0	0.0%	1	0.6%		0	0.0%	1	1.0%	
Sexual dysfunction	0	0.0%	1	0.6%		0	0.0%	1	1.0%	
Urinary complication	0	0.0%	3	1.8%		0	0.0%	3	3.1%	

*PAS* previous abdominal surgery, *CVC* central venous catheter

## Discussion

In the present study, we compared the perioperative outcomes, postoperative pathological outcomes, and oncological outcomes of preoperative CCRT and robotic-assisted rectal surgery between PAS and non-PAS patients with locally advanced rectal adenocarcinoma. To minimize selection bias, we performed PSM between the patient groups and found no significant between-group differences in perioperative outcomes, postoperative pathological outcomes, and oncological outcomes both before and after PSM.

Before PSM, the PAS group included significantly more women than men (62.9% vs. 37.1%, *P* = 0.006), consistent with a previous study [18]. This significant difference was due to common gynecologic surgical procedures, including abdominal total hysterectomy, cesarean section, and cesarean section, being commonly employed in female patients. Here, 20 (90.9%) of 22 female PAS patients underwent the aforementioned procedures, which may have caused adhesions in the pelvic cavity and increased the difficulty of robotic-assisted rectal surgery and the risk of perioperative complications. Although the median console time was significantly shorter in PAS patients than in non-PAS patients before PSM, the median operation time was not significantly different between the two groups before or after PAM. These findings are consistent with those reported previously [18]. Studies evaluating the impact of PAS on laparoscopic colorectal surgical outcomes [14–18] have found no significant between-group differences in the operation time. However, in a large case-control study on 756 patients [19], Zeng et al. found that PAS was associated with longer operation time (220 vs. 200 min, *P* = 0.002). In the present study, other perioperative outcomes were similar between the groups, consistent with previous results [18].

Studies on the impact of PAS on the outcomes of laparoscopic colorectal surgery [14–18] have reported no significant difference in complication rate [18]. Similarly, in the current study, the overall complication rates and specifically anastomosis leakage and postoperative ileus rates did not differ significantly between the groups before or after PSM corroborating the previous results [18].

We also evaluated the impact of PAS on postoperative pathological and oncological outcomes and found nonsignificant between-group differences before and after PSM. Intraabdominal adhesion may increase surgical difficulty, and most intraabdominal adhesions may result from abdominal surgical procedures. Therefore, PAS could also result in surgical difficulties. Kang et al. [26] reported that technical difficulties during laparoscopic surgery for CRC could negatively affect oncologic safety. By contrast, Lee et al. [17] demonstrated that technical difficulties due to intraperitoneal adhesions do not impede the oncologic safety of patients with CRC undergoing laparoscopic surgery. These findings are consistent with those of Zeng et al. [19], who found nonsignificant differences in the 3-year DFS and 3-year OS in their case-control study.

To the best of our knowledge, few studies have evaluated the impact of PAS on robotic-assisted colorectal surgical outcomes [18, 21]. Park et al. [18] investigated the impact of PAS on robotic-assisted colorectal surgery in 238 patients with CRC (87 patients with colon cancer and 151 patients with rectal cancer). In the present study, we evaluated the impact of PAS on robotic-assisted rectal surgery in 203 patients with rectal cancer, all of whom underwent preoperative CCRT. By contrast, in the aforementioned study [18], only 29 (12.2%) patients underwent preoperative CCRT. Moreover, in the present study, 140 (68.9%) patients had stage III disease, whereas only 89 (37.4%) patients had stage III disease in the aforementioned study [18]. However, our perioperative outcomes, namely, operation time, estimated blood loss, and time of resuming soft diet, were comparable with those of the aforementioned study [18]. Hu et al. [21] conducted a meta-analysis to evaluate the effect of PAS on perioperative recovery outcomes of robotic-assisted colorectal surgery; the authors determined an objective conclusion by comparing perioperative outcomes and provided level I evidence for clinical decision-making [21].

The current study has several limitations. First, this was a single-center retrospective study with a small sample size (*n* = 203 patients, including 35 PAS and 168 non-PAS patients). However, we further used PSM to match the compatible groups and reduce the potential selection bias. Second, the retrospective nature of this study prevented evaluation of the actual severity of intraabdominal adhesions, which may affect the difficulties of robotic-assisted rectal surgical procedures. Third, the follow-up interval was relatively shorter, with a median follow-up of 28 months; thus, only the 1- and 3-year oncological outcomes were documented. Fourth, we did not evaluate the postoperative outcomes of urinary, sexual functions, or anal functions. Fifth, in the present study, we only investigated the impact of PAS on robotic-assisted rectal surgery in patients with rectal cancer, and we did not evaluate whether PAS might have a different effect on postoperative outcomes in patients with rectal versus colon cancer. Sixth, because this was a single-center retrospective study, we did not have the sizes of the previous incisions in the chart records. The sizes of the previous incisions may affect the intraabdominal adhesions. Seventh, the covariates used in PSM were already nonsignificant except of gender, so the matching effect will be limited. Eighth, we did not perform a multiple testing correction procedure. Nonetheless, it seems unlikely that the *P* values will change much.

## Conclusions

In the present study, PAS and non-PAS patients demonstrated similar perioperative outcomes and short-term oncological outcomes of robotic-assisted rectal surgery, without any effect on the overall complication rate. Therefore, robotic-assisted surgery may be safe and feasible in PAS patients with locally advanced rectal adenocarcinoma undergoing preoperative CCRT. However, further researches including a longer follow-up duration investigating the long-term oncological outcomes are warranted. Moreover, prospective randomized clinical trials are required for validating the present results.

## Data Availability

All data used to support these findings are included in the article.

## Abbreviations

AJCC: American Joint Commission on Cancer
ASA: American Society of Anesthesiologists
BMI: Body mass index
CCRT: Concurrent chemoradiotherapy
CEA: Carcinoembryonic antigen
CRC: Colorectal cancer
CRM: Circumferential resection margin
CT: Computed tomography
DFS: Disease-free survival
DRM: Distal resection margin
LARC: Locally advanced rectal cancer
LCRT: Long-course radiotherapy
MRI: Magnetic resonance imaging
OS: Overall survival
PAS: Pervious abdominal surgery
PSM: Propensity score matching
TME: Total mesorectal excision
TRG: Tumor regression grade
VAS: Visual analog scale
